# Foreign Direct Investment in oil-abundant countries: The role of institutions

**DOI:** 10.1371/journal.pone.0215650

**Published:** 2019-04-23

**Authors:** Federico Carril-Caccia, Juliette Milgram-Baleix, Jordi Paniagua

**Affiliations:** 1 Department of Economic Theory and History, University of Granada, Granada, Spain; 2 Department of Economic Analysis and Political Economy, University of Seville, Seville, Spain; 3 Department of Economic Structure, University of Valencia, Valencia, Spain; Universidad de Castilla-La Mancha, SPAIN

## Abstract

The present work reassesses the impact of good governance and democracy on Foreign Direct Investment (FDI) in oil-abundant countries. To this end, we estimate the effect of host countries’ institutions on greenfield FDI, using a gravity equation for a dataset that covers 182 countries during 2003-2012. Our findings confirm that compliance to rule of law, lack of corruption, political stability and democracy could boost new FDI links through the extensive margin. Our results could not rule out the “oil curse”, meaning that oil producers attract fewer new greenfield projects than similar countries without oil. Unlike other studies, we show that the impact of institutions is not necessarily undermined by the presence of natural resources.

## Introduction

Recent decades have witnessed ups and downs in oil prices, provoking economic and social instability in oil-abundant countries, serving as a reminder of how important it might be to diversify their economies. Foreign Direct Investment (FDI) could improve these countries’ development as it can bring new technologies, broaden access to new markets through exports, and diversify economic activity. According to [1], FDI is one of the main pillars of development strategies in resource-rich countries as it can also help natural-resource-based activities to foster growth through new skills and technologies.

Natural resources can be broadly divided into two main categories; point and diffuse sources. Examples of the former are oil and minerals. These types of resources are characterized by their specific localization, and ownership of production and revenues are concentrated among few agents. Alternatively, agriculture or forestry are more likely to be more dispersed through the economy. Abundance in point-source natural resources is extensively associated with countries’ low economic performance [2, 3]. The natural-resource curse entails low economic growth through lack of trade of manufactured goods, low institutional quality characterized by predatory institutions and undesirable allocation of resources in favour of rent seeking and in detriment of human capital formation or investment in productive activities (e.g. [4–7]). Successfully managing resource wealth is an economic and political process that requires private investment and judicious policies to hedge adverse impacts [8]. Research has identified both a positive e.g. [9, 10] and a negative e.g. [11, 12] relationship between FDI & natural-resource endowments.

Each natural resource is exposed to different price fluctuations and entails different extractive costs and technology and as a consequence different investments. Therefore, it seems preferable to focus, in empirical studies, on homogeneous resources, even if some mechanisms described in the theoretical literature between point-source resources, Institutions and FDI are common to oil and minerals. According to the World Bank Development Indicators, oil rents represented 60% of natural-resource rents at the world level during the period 2003-2012. Due to its economic relevance, this study focuses on oil.

The aim of this paper is to tackle the relationship between institutional quality and FDI in oil-abundant countries. This issue has received little attention, with several notable exceptions that make the issue even more puzzling. Indeed, existing evidence suggests that endowment in oil, fuels or minerals weakens the positive impact that democracy and institutional quality have on FDI (e.g. [11, 13]). These findings contrast with the extensive literature that highlights the relevance of good governance for attracting FDI (e.g. [14, 15]) or that suggests that the natural-resource curse could be turned into a blessing through institutional improvements (e.g. [16]).

A specific contribution to the subject of the impact of point-source natural resources on the institution-FDI nexus is to provide solid empirical evidence in a broader panel setting. Previous studies have two shortcomings: they usually focus on single countries or a reduced subset and analyse aggregate FDI inflows, regardless of the bilateral nature of FDI. At the aggregate level, FDI inflows could influence institutional quality with the subsequent potential endogeneity bias. To hedge these limitations, we estimate bilateral greenfield FDI flows for 182 countries during 2003-2012 by means of the gravity equation. Greenfield investment represents more than half of the world’s FDI projects and 72% of the total FDI projects received by developing countries. This kind of investment signifies a notable entry of investments and knowledge for the recipient country. The world’s total number of FDI projects is calculated as the sum of greenfield investment and merger and acquisitions projects. Shares are calculated by the authors based on the annex Tables 11 and 22 from [17].

Unlike other studies, we delve into the factors that determine the creation of new investment links at the country level (extensive margin). This allows us to offer a better understanding of the question of whether there is an oil curse on FDI, and which aspects of good governance matter most for attracting FDI in oil-producing countries.

Our results suggest that rule of law, lack of corruption, political stability and democracy are relevant determinants of new greenfield investment projects. Results also validate the hypothesis of an “oil curse” on new investment linkages, but do not support the idea that countries’ oil production undermines the positive impact of good governance on greenfield investment. In fact, the benefits from institutional improvements increase with the relevance of oil rents in the host economy. This finding is confirmed by the robustness analysis which accounts for omitted variables, causality between FDI, institutions and oil production, and alternative measures of oil abundance.

Based on this analysis, we illustrate how institutional reforms would affect a country’s capacity for attracting greenfield FDI for different levels of oil production. Furthermore, we infer the level of institutional quality that would allow, all else being equal, a country to overcome the oil curse on FDI.

The remainder of the paper proceeds as follows. The next section details how the abundance of natural resources may interfere in the institution-FDI nexus. Section III describes the methodology and data used. Section IV presents the results, which are followed by a sensitivity and robustness analysis in Section V. Finally, Section VI concludes.

## Theoretical and empirical review

### Does the quality of institutions attract FDI?

There are several reasons why the quality of institutions matters for FDI. The economic growth literature suggests that better institutions may generate more economic growth through better incentives to invest and more efficient allocation of resources [18]. In addition, high-quality institutions are also expected to reduce information asymmetries, providing information about market conditions, goods and participants, which in turn can encourage (domestic and foreign) investment in the country [19]. In contrast, a “bad” institutional environment may increase the cost of doing business either by uncertainty brought about by political instability or corruption and poor compliance to the rule of law.

Even if the widespread conviction is that good governance tends to attract FDI, theoretical and empirical studies that examine more precise aspects of institutions draw a more ambiguous relationship. Most studies confirm that political risks deter investment from multinational enterprises (MNEs) for different sets of countries e.g. [14, 15, 20–26] while others find no evidence linking political risks to FDI e.g. [27–30] and [10]. However, [31] and [32] report a discrepant negative relationship between stability and FDI. The expected effects of corruption on FDI are particularly controversial. At first glance, corruption clearly increases the transactional costs of foreign firms and thus should deter FDI. This conclusion is validated by several studies e.g. [9, 10, 20, 21, 24, 33–36] and [37]. Yet, corruption is also seen as the “helping hand”, at least at the firm level and despite the negative aggregate outcome on growth (see [38–40]). Indeed, in an institutional framework characterised by inefficient bureaucracy, these illegal practices may also be a way to circumvent an inefficient administration or influence government policies to the benefit of the MNE [36, 41].

### FDI and institutions quality in the context of resource-rich countries

When point-source natural resources are at stake, MNEs may be encouraged by lower institutional quality since in this way they are able to appropriate a larger share of its rents and enjoy greater bargaining power [12, 42]. In this regard, [43] states that countries rich in this type of natural resources could attract a larger share of FDI by offering cheap access, even if there is a high expropriation risk. The author suggests that the penalty for host countries’ governments lessens, as the value of foreign assets in the sector increases and the royalties for exploiting natural resources paid by MNEs decrease. [13] conclude that MNEs always exhibit institutional risk aversion, although investment returns in countries with low capital intensities but with abundance in natural resources may outweigh the costs associated with institutional risk. Nevertheless, MNEs operating in this sector are constrained by the limited availability of the natural resources, converting this specificity into a pre-condition of their location choice, regardless of the institutional framework [42, 44].

Along the same lines, [45] show that, for FDI originating from developing countries, the negative impact of “bad” institutions on FDI inflows is lower when the host country is abundant in point-source natural resources (For a survey of the motivations of FDI from emerging countries, see for instance [46]). [44, 47] and [13] find similar results for Chinese outward FDI, explaining that Chinese FDI is not attracted by bad institutions *per se* but rather by natural resources that correlate with bad institutions. In a similar vein, [48] report that property rights do not have a significant impact on FDI directed towards the primary sector.

### FDI and the political system in the context of resource-rich countries

Institutions are in turn shaped by the political system, namely, the degree of democracy or autocracy [49]. Democracies tend to be more predictable and make their preferences clear [50], thus reducing investment uncertainty. Additionally, democracies may be accompanied by countries’ openness to the world economy [51]. The lack of democracy boosts social tensions that increase the likelihood of bringing severe political and social crisis to a country [52]. Similarly, [11, 14, 28, 50, 51, 53, 54] and [55] point out a positive relationship between democracy and FDI. In contrast, [31, 38, 56] and [57] evidence a negative relationship, while others fail to find a significant effect [29, 39, 58] and [59].

Some characteristics of democracies, such as changes of governments and policies, may be seen as drawbacks for MNEs. More precisely, in countries abundant in natural resources, autocracies may offer more advantages than disadvantages to those foreign firms interested in investing in the resource sector for rent-seeking motives. This is mainly due to the fact that point-source natural resources, and particularly oil, are controlled by local authorities. [11] suggest that MNEs in the extractive industry wish to avoid frequent changes of governments, since governments that have long-term stability favour closer ties. Moreover, [38] argue that when investment seeks to access natural resources, MNEs may prefer slight civil repression.

The empirical validations of the above hypotheses are scarce. [11] find that democracy has a positive impact for FDI but the share of mineral and oil in total exports undermines the positive effect of democracy on FDI. Similarly, [50] find that media freedom has a negative influence on FDI that outweighs the positive impact of other democratic attributes when both natural resources, measured by the share of mineral and oil in total exports, and income inequality are high. [55] report a strong link between democracy and FDI among all industries except mining and oil and gas extraction.

### Can good institutions cancel out the FDI resource curse?

The above reviewed research is linked to the extensive strand of the literature studying the negative effects of substantial natural-resource endowment on countries’ performance. This paradoxical phenomenon that may turn the “blessing” of natural resources into a “curse” is also often referred to as the Dutch disease. Resource discoveries may have a negative effect on growth since it generates a large increase in exports which in turn leads to an appreciation of the local currency. This makes the country’s exports less competitive at world prices, and thereby crowds out investments in non-natural-resource tradable sectors. Productive activities that boost growth decline in favour of the natural-resource sector for rent-seeking purposes [4, 7]. Natural-resource abundance is also likely to favour bad institutions in detriment of pro-growth behaviour. The rents provided by the exploitation of natural resources are easily appropriated generating a “rentier effect” [3, 6, 12, 45]. Moreover, revenues from the export of fuels and minerals allow governments to quieten critics and avoid accountability pressures [2]. Natural-resource abundance breeds corruption [45] and raises expropriation risks [43]. Furthermore, growth collapses due to a high dependence on natural-resource rents and international price volatility, and domestic struggles for the control of natural resources, can result in social unrest and violence [5].

However, point-source natural resources do not necessarily imply countries’ low economic performance. In this regard, Norway is the paradigmatic example. [1, 16] and [60] argue that good governance could potentially turn the natural-resources curse into a blessing by investing the capital brought by natural resources into productive activities or promoting knowledge-intensive economic activities, hence promoting economic growth.

The mechanisms described above may have a direct effect on FDI. Indeed, FDI inflows are attracted by high expected returns in the resource sector, and decrease in the non-resource sector. The likelihood of an overall negative effect is high and referred to as a “FDI-resource curse” [20]. Other indirect effects are also liable to deter FDI. For instance, macroeconomic instability could increase since the volatility of the exchange rate is expected to rise due to the booms and busts that characterise natural-resource prices [7] and due to the lower trade diversification makes a country more vulnerable to external shocks. This adverse context may deter FDI. [11] also argue that FDI in natural resources is expected to stagger after the initial phase since less capital is needed to continue the exploration that is needed to start it.

Surprisingly, very few studies back this hypothesis. [12] show, for Dutch FDI into 183 host countries that FDI flows to the natural-resource sector do not compensate for the disinvestments in the non-resource sector. Similar results are reached by [30] for 16 Middle East and North Africa (MENA) countries and by [61] and [24] for Gulf Cooperation Council countries. Nonetheless, the majority of studies focusing on small datasets acknowledge that the availability of point-source natural resources has a positive and significant effect on FDI in developing countries. See for instance [62] for 53 African countries, [33] for 22 countries in Sub-Saharan Africa (SSA), [21] for 16 Arab economies, [9] using a panel of 36 developing countries, [25] for 108 autocratic countries and [63] for 22 Sub-Saharan African countries. However, for larger datasets the evidence is scant and mixed. [45] find a non-significant effect of resources on bilateral FDI flows (Their dataset includes 60 developing and 22 developed economies). [11] and [20] conclude that point-source natural resources have an adverse effect on FDI. [11] study a sample of 112 developing countries and [20] focuses on 99 developing countries. In contrast, [10], for a sample of 125 developing countries, find that point-source natural resources foster inward FDI.

[12] address the question of the role of quality institutions as a mediator in the natural resources-FDI nexus but reject the hypothesis of a significant influence. [20] also confirms that institutional quality may be able to reduce, but not fully cancel, the effect of natural resources on FDI. [53] demonstrates that FDI is positively moderated by the accumulation of democratic capital, and shows that the association between FDI and democracy is not affected by resource dependence.

## Methodology and data overview

### Empirical model

Unlike most of the studies reviewed, our empirical model explores the bilateral dimension of FDI. As demonstrated by [64], traditional gravity variables are better candidates for explaining FDI activity than merely host-country characteristics. Another decision regards the choice of the dependent variable. Most studies focus on the amount of FDI flows or FDI stocks, measuring therefore the intensive margin of FDI. Very few are able to measure the extensive margin of FDI since they work with macro data. Indeed, there are several advantages to working on the number of projects rather than flows. First, due to the existence of fixed FDI costs, selection of firms into FDI is limited [65], in analogy with the export behaviour underlined by [66]. Hence, as long as the institutional framework reduces or increases these sunk investing costs, the quality of institutions is more likely to influence the preliminary decision to develop new projects of investments [67] than the invested amount. Second, flows are sometimes dependent on one or two large investment projects, especially in relatively small countries, so relying on the stocks of FDI may be misleading [34, 68].

Following these arguments, we estimate the effect of several indicators of host-country institutions on the number of bilateral greenfield investment projects, using a standard gravity equation. The gravity equation is the workhorse of empirical studies that analyse international economic flows. The gravity model was first developed to study the determinants of bilateral trade flows (for an overview see [69] and [70]). This framework offers several advantages, which have contributed to its popularity in examining trade, capital, migration and tourism flows. First, the gravity equation for trade has been founded on solid theoretical grounds since [71] and [72]. Following trade developments, studies such as [73–75] and [76, 77] have developed theoretical models that result in empirical equations for the case of FDI. Second, empirical advances in the estimation of the gravity equation have led to estimations that minimize known biases such as the abundance of zeros and the heteroscedasticity in the error term [78]. Third, the gravity equation delivers a flexible toolkit to estimate our variables of interest while controlling for both observable and unobservable confounding factors. Most notable is the inclusion of multilateral resistance terms that control for third country effects.

We are interested in estimating the joint effect of domestic institutions and oil endowments on greenfield FDI projects. The work of [57], which studies the role of domestic legal rights and democracy in advanced and developed countries, provides a starting point that we extend in several ways. Firstly, we frame our study on the economic impact of institutions rather than on business ethics. [57] were interested in the ethical implications of FDI and in so doing looked only at the effect of the host development level on the marginal effect of democracy on FDI. Their golden rule of FDI ethics considered the incentives of becoming democratic in least developed countries. Therefore, the role of natural-resource endowments, namely oil, was left out of their framework. Our focus on oil rents is an additional channel proposed by the economics literature. Secondly, we extend the institutional variables by adding lack of corruption and political stability. Thirdly, we perform a battery of robustness checks to support our analysis. To this end, we estimate the following specification:

$$\begin{array}{c} FDI_{ijt}=e^{\left(\begin{array}{c} \beta_{1}ln\left(GDP_{it}xGDP_{jt}\right)+\beta_{2}ln\left(Distance_{ij}\right)+\beta_{3}BORDER_{ij}\\ +\beta_{4}LANGUAGE_{ij}+\beta_{5}COLONY_{ij}+\beta_{6}SMCNTRY_{ij}+\beta_{7}Religion_{ij}\\ +\beta_{8}FTA_{ijt}+\beta_{9}BIT_{ijt}+\beta_{10}Inst_{jt}+\beta_{11}Inst_{jt}xOILP_{j}+FE \end{array}\right)}+\epsilon_{ijt} \end{array}$$ (1)

where *i*, *j* and *t* stand respectively for the source, the host country and the year. *FDI*_*ijt*_ is the number of greenfield projects undertaken by firms from country *i* in the host country *j*, in year *t*; *GDP*_*it*_ and *GDP*_*jt*_ are the GDPs of home and host countries, respectively; *Distance*_*ij*_ is the distance in kilometres between country capitals; *BORDER*_*ij*_ is a dummy that indicates whether a pair of countries share a common border; *LANGUAGE*_*ij*_ takes positive value if both countries share the same official language; *COLONY*_*ij*_ is set to one if the two countries have ever had a colonial link; *Religion*_*ij*_ is a composite index that measures the religious affinity between country pairs with values from zero to one; *SMCNTRY*_*ij*_ indicates if both countries were part of the same country in the past; *FTA*_*ijt*_ is a dummy that indicates whether both countries have a free trade agreement in force; *BIT*_*ijt*_ is a dummy that takes a value of one if the country pair has a bilateral investment treaty in force. Next, *Inst*_*jt*_ stands for institutions, *OILP*_*j*_ is a dummy that represents significant oil producers. Lastly, FE stands for the host and home country, and year fixed effects (respectively, λ_*i*_, λ_*j*_, λ_*t*_) and *ϵ*_*ijt*_ represents the stochastic error term. Following [78], we account for null flows in bilateral FDI data by using a Poisson Pseudo-Maximum Likelihood estimator.

Additionally, Eq 1 is modified to address the impact of oil production on FDI and how different levels of oil production alter the FDI-institutions nexus. In particular we replaced *Inst*_*jt*_*xOILP*_*j*_ by the share of oil rents over GDP (*Oilrents*_*jt*_) and interacted the oil measure with institutional quality. Due to the high correlation between institutional variables (see Table 1), namely rule of law, lack of corruption, political stability and democracy, they are estimated separately.

**Table 1:** Correlation matrix.

	Greenfield Investment projects	Oil rents	Rule of law	Lack of corruption	Political stability
Oil rents	-0.119***	1			
Rule of law	0.278***	-0.271***	1		
Lack of corruption	0.242***	-0.277***	0.953***	1	
Political stability	0.095***	-0.149***	0.785***	0.757***	1
Democracy	0.118***	-0.549***	0.464***	0.434***	0.288***

Note: Authors’ own calculation.

* *p* < 0.10,

** *p* < 0.05,

*** *p* < 0.01

### Data overview

Our analysis covers 182 countries during 2003-2012. Since the dataset contains many zeros (70% of the potential observations are zero), we have followed the proposal of [79] to implement an efficient gravity dataset. Basically, we consider zeros only those country-pairs with a track record of investment in some year. Using a panel (with variation across country-pairs and time) allows us to explore a better identification strategy. Additionally, [80] argue that the PPML estimator is appropriate for short panel gravity data, such as the one used in this study.

Statistics for the variables used are available in Table 2. The countries included in the sample are reported in Table A in the S1 Appendix. Data for bilateral greenfield investments are gathered from FDI Markets. GDP (in constant year 2000 USD) is retrieved from the World Bank. Distance, common language, colony, and border are from the CEPII dataset and religious affinity is obtained from the CIA World Factbook. BIT variable is constructed based on UNCTAD’s International Investment Agreements database and FTA comes from [81].

**Table 2:** Descriptive statistics.

	Mean	Std. Dev.	Min	Max
FDI	1.77	8.26	0	319
*ln*(*GDP*_*it*_*xGDP*_*jt*_)	27.09	1.51	20.12	30.40
*ln*(*Distance*)	8.31	1.00	4.09	9.88
*BORDER*	0.06	0.24	0	1
*LANGUAGE*	0.16	0.37	0	1
*COLONY*	0.05	0.21	0	1
*SMCNTRY*	0.02	0.14	0	1
*Religion*	0.33	0.32	0	1
*FTA*	0.26	0.44	0	1
*BIT*	0.42	0.49	0	1
*OILP*	0.20	0.40	0	1
*OilRents*	5.67	13.61	0	343.74
*Ruleoflaw*	3.85	1.01	1.71	5.67
*Lackofcorruption*	3.12	1.06	1.11	5.48
*Politicalstability*	4.28	0.94	1.14	5.99
*Democracy*	14.64	6.50	0	20

Note: authors’ own calculations.

To measure institutional quality, we consider three different indices: *rule of law*, *lack of corruption* and *political stability* from the World Bank’s Worldwide Governance Indicators. The choice of the source of these indicators is based on their wide country coverage and use in previous works (e.g. [44, 45, 67, 82]). These variables range approximately from -2.5 to +2.5 [83]. To facilitate interpretation in the econometric analysis, we convert them into non-negative values equal or larger than 1. Higher values suggest respectively better rule of law, less corruption and a more stable political environment.

Regarding countries’ political systems, we use the *Polity2* index from the Polity IV dataset retrieved from Systemic Peace [84]. The *Polity2* index, which we name Democracy, ranges from -10 (full autocracy) to 10 (full democracy). Again, for the econometric analysis this variable is re-scaled so that it takes values between 0 and 20, 0 representing a full autocracy, and 20 a full democracy.

Democracy and autocracy are measured independently without sharing categories in common. The degree of both are based on how a country scores in: competitiveness of executive recruitment, openness of executive recruitment, constraint on chief executives and competitiveness of political participation. Nevertheless, the items that define these variables are different. For instance, when measuring the openness of executive recruitment, a democratic country will score one point if elections are held, or one point as autocracy if chief executives are determined by hereditary succession. The *Polity2* score is computed by subtracting the score obtained by the index autocracy score from the democracy score. In this way, the *Polity2* index enables us to take into account intermediate situations between full autocracy and democracy. For instance, according to the classification used by Systemic Peace, Saudi Arabia is an autocracy, Egypt is a closed anocracy, Algeria is an open anocracy, Lebanon a democracy and Israel a full democracy [84]. We consider this measure as appropriate since it is based on objective information and because the political system of a country is not a one-dimensional characteristic, but probably includes several dimensions [38]. Moreover, the *Polity2* index has been extensively used in the literature e.g. [11, 31, 53].

To identify the countries in which oil production represents a significant share of domestic economy, we use the indicator named Oil Rents from the World Bank. It represents the difference between the value of crude oil production at world prices and total costs of production over GDP. We consider oil production as relevant for a country when Oil Rents represent at least 7.65% of the country’s GDP in at least one year; this threshold stands for the top quintile of countries in our sample. In this way, our sample is divided into countries in which oil production does have a relevant role throughout our period, and those in which its relevance is anecdotic and limited.

## Results

### The FDI-institutions nexus

Results from our baseline model are reported in Table 3. As usual in the literature, the gravity equation performs well, explaining more than 80% of the variation of the dependent variables. The results for distance and FTA support the hypothesis of complementarity between trade and FDI. In addition, the lack of significance of the combined home and host countries’ economic sizes indicates that greenfield investment projects are driven by fragmentation of production. The factor-proportion theory predicts the host country’s demand to increase the likelihood of production fragmentation, while for the home countries the opposite is expected [74]. Likewise, sharing a common border deters FDI, implying that MNEs may prefer to serve neighbour countries through exports. As expected, sharing a language, religious affinities and historical ties have a positive impact on the number of greenfield projects consistent with a reduction of sunk costs. Finally, BIT lacks significance. This finding is not surprising, as previous studies indicate that the significance of BIT depends on the quality of interstate relations and host countries’ institutional quality [85], the level of development of signing countries [86], intensity of bilateral FDI flows [87] or the sector of investment [36].

**Table 3:** The FDI-institution nexus.

FDI	Rule of law	Lack of corruption	Political stability	Democracy
Extensive margin	(1)	(2)	(3)	(4)
*ln*(*GDP*_*it*_*xGDP*_*jt*_)	-0.078	-0.075	-0.030	-0.084
	(0.18)	(0.18)	(0.17)	(0.18)
*ln*(*Distance*_*ij*_)	-0.389***	-0.389***	-0.388***	-0.390***
	(0.03)	(0.03)	(0.03)	(0.03)
*BORDER*_*ij*_	-0.135*	-0.135*	-0.135*	-0.128
	(0.08)	(0.08)	(0.08)	(0.08)
*LANGUAGE*_*ij*_	0.508***	0.508***	0.508***	0.502***
	(0.06)	(0.06)	(0.06)	(0.06)
*COLONY*_*ij*_	0.596***	0.595***	0.595***	0.586***
	(0.08)	(0.08)	(0.08)	(0.08)
*SMCNTRY*_*ij*_	0.566***	0.566***	0.566***	0.568***
	(0.15)	(0.15)	(0.15)	(0.16)
*Religion*_*ij*_	0.389***	0.388***	0.389***	0.409***
	(0.13)	(0.13)	(0.13)	(0.13)
*FTA*_*ijt*_	0.195***	0.193***	0.196***	0.187***
	(0.06)	(0.06)	(0.06)	(0.06)
*BIT*_*ijt*_	-0.028	-0.029	-0.027	-0.030
	(0.04)	(0.04)	(0.04)	(0.04)
*Inst*_*jt*_	0.112	-0.053	0.309***	0.014*
	(0.15)	(0.09)	(0.06)	(0.01)
*Inst*_*jt*_*xOILP*_*j*_	0.232	0.420***	-0.198*	0.044*
	(0.25)	(0.15)	(0.10)	(0.02)
Fixed effects	Home, host and year fixed effects			
Observations	39151	39151	39118	37153
*R*^2^	0.844	0.845	0.847	0.845

Robust standard errors in parentheses

* *p* < 0.10,

** *p* < 0.05,

*** *p* < 0.01

Concerning the impact of institutions on the number of greenfield investment projects, *Inst*_*jt*_ and *Inst*_*jt*_*xOILP*_*j*_ respectively distinguish between non-significant oil producers and significant oil producers. For both groups of countries, rule of law does not play a relevant role in explaining the capacity to attract greenfield investment. Similar to the case of rule of law, the lack of corruption in non-oil producers is not significant. On the other hand, for countries that are significant oil producers, reducing corruption would increase a country’s probability of receiving FDI. This last finding supports the “grabbing hand” hypothesis and is in contrast to the findings from [38] and [40], who find a positive relationship between the level of corruption and inward FDI. In addition, this result echoes those from [67], which show that the level of corruption reduces a country’s probability of receiving FDI, or the results of [41], who find that corruption affects new entrants in a more obvious negative way than incumbent ones.

Political stability appears to be crucial to increase the number of foreign projects for both group of countries. Political instability, that is, politically motivated violence and terrorism, might not be easily compensated for by countries’ oil endowment. Nevertheless, the overall positive effect of stability on greenfield investment is lower for countries that are significant oil producers. As these natural resources are concentrated only in a few countries, this lower impact might support the view of a higher tolerance by MNEs to instability [13, 42, 44, 47].

Overall, the degree of democracy enhances countries’ capacity to attract new projects. In addition, the impact of democracy is larger for oil producers than for other countries, as in [50], but in contrast to [11].

Yet, unlike most studies focusing on aggregate FDI, we explain bilateral projects. This enables us to control for country pair characteristics such as cultural and geographical and economic distance. These differences might explain part of the divergence with some of the findings reported by previous studies that use unilateral FDI flows (e.g. [31, 38, 40]) and the few studies using bilateral data for FDI to study the nexus between institutions and FDI intensive margin, which usually find a less significant effect [22, 57, 64], or a positive effect of the quality of institutions when they focus on a smaller country sample e.g. [39, 88, 89] and [15].

### Oil production and institutions

In this section, we further inquire into the role of natural resources in the Institution-FDI relationship. As we have already mentioned, Table 3 reports the average expected impact of institutions for significant oil producers However, the dummy which represent significant oil producers (*OILP*_*j*_) might also capture the potential effect of oil endowment on FDI. We now look into how different levels of oil production affect FDI and alter the FDI-Institutions nexus. Table 4 shows the impact of oil rents over GDP (*OilRents*_*jt*_) on FDI, the expected impact of institutions (*Inst*_*jt*_) and the impact of the combined effect of institutions and oil rents (*OilRents*_*jt*_*xInst*_*jt*_), which respectively have associated coefficients *β*_10_, *β*_11_ and *β*_12_.

**Table 4:** Institutions and oil rents over GDP.

FDI	Rule of law	Lack of corruption	Political stability	Democracy
Extensive margin	(1)	(2)	(3)	(4)
*OilRents*_*jt*_	-0.056***	-0.042***	-0.019*	-0.001
	(0.01)	(0.01)	(0.01)	(0.01)
*Inst*_*jt*_	0.116	-0.038	0.243***	0.022***
	(0.13)	(0.08)	(0.06)	(0.01)
*OilRents*_*jt*_*xInst*_*jt*_	0.017***	0.016***	0.005**	0.000
	(0.00)	(0.00)	(0.00)	(0.00)
Fixed effects	Home, host and year fixed effects			
Control variables	YES	YES	YES	YES
Observations	36037	36037	36004	34920
*R*^2^	0.846	0.846	0.848	0.847

Robust standard errors in parentheses

* *p* < 0.10,

** *p* < 0.05,

*** *p* < 0.01

Coefficients of control variables are available on request.

#### Is there an FDI-resource curse?

Results support the hypothesis of an “oil curse” on FDI’s extensive margin: that is, the higher the oil production, the lower the number of greenfield investment projects. Again, our results are in line with those of [11, 20] and [12], who claim the existence of an oil curse on the capacity of attracting FDI. According to the estimates, one percentage point increase in the share of oil rents over GDP can reduce the number of projects by nearly 3% on average (Table 4).

#### Does the presence of oil undermine the effect of institutions on FDI?

The effect of institutions (i.e. ${\hat{\beta }}_{11}$), regardless of the level of production is not clear-cut. Only political stability and democracy would improve the extensive margin while rule of law and lack of corruption have no effect. Alternatively, we register positive and significant coefficients for *Inst*_*jt*_*xOilRents*_*jt*_ (${\hat{\beta }}_{12}$), indicating that the importance of natural resources magnifies the impact of institutions on FDI. The exception is the coefficient associated with *Democracy*_*jt*_*xOilRents*_*jt*_, which is null and non-significant. Thus, these results confirm again that institutions would, on average, have a positive impact for countries that are significant oil producers. In addition, they show that the gains to be obtained from improvements in institutional quality, and to a lesser extent democracy, are exacerbated (and not undermined) by countries’ oil production.

These results contradict those of [11] who acknowledge that the relationship between FDI and democracy depends negatively on the “size” of natural resources measured by the share of fuel and minerals in total merchandise exports. However, the results are in line with [20] for institutional quality.

#### How large are the benefits to be obtained from better institutions?

As in [11], we assess the magnitude of the benefits, in terms of new greenfield investments, that could be expected from an improvement in institutions. Based on the estimates reported in Table 4, we calculate the percentage change in the number of greenfield projects as a consequence of a one-point change in the institutional indicator (*Inst*) given the average level of oil production (*OilRents*):

$$\begin{array}{c} \frac{\partial ln(FDI)}{\partial Inst}={\hat{\beta }}_{11}+{\hat{\beta }}_{12}\bar{Oil} \end{array}$$ (2)

where ($\bar{Oil}$) is the average level of oil rents over GDP during the period 2003-2012. Although the estimated equation is a non-linear, its interpretation is equivalent to a log-linearized equation [78].

Table 5 illustrates the expected average change in the number of greenfield projects for countries belonging to different percentiles according to their share of oil rents over GDP. Oil producers belonging to the top percentiles in terms of Oil rents would greatly benefit from improving rule of law and reducing corruption. For a country like Azerbaijan, a one-point improvement in the rule of law and lack of corruption indices would augment the number of greenfield projects by 65% and 46% respectively. This would mean rallying to a level similar to that registered by Croatia. However, the gains from reducing corruption are not clear for all countries. For those countries in which oil rents are below the sample’s median (1.35%), reducing corruption can deter new greenfield projects. The gains to be obtained from advancement towards political stability are also substantial while lower than for the above-mentioned indicators. For a country that does not produce oil (e.g. Paraguay), a progress of one point would translate into an increment of 24% in the number of projects, and similar gains are expected for those countries close to the sample’s median. Alternatively, for a country like Syria, the gains would be of 35%.

**Table 5:** Impact of institutional reform given the level of oil rents over GDP.

Percentile of Oil rents	Oil rents	As in	Rule of law	Lack of corruption	Political stability
10	0.01%	Jordan	11.62%	-3.78%	24.31%
25	0.17%	Philippines	11.90%	-3.52%	24.39%
50	1.35%	Myanmar (Burma)	13.90%	-1.63%	24.98%
75	7.07%	Suriname	23.61%	7.50%	27.83%
90	22.27%	Syria	49.46%	31.83%	35.43%
95	31.43%	Azerbaijan	65.04%	46.49%	40.02%
100	45.86%	Saudi Arabia	89.56%	69.58%	47.23%
Average	8.74%	Papua New Guinea	26.46%	10.18%	28.67%

Note: Authors’ own calculations, based on estimates from Table 4 and the average level of oil rents over GDP during the period 2003-2012 for those countries in which oil rents are higher than 0%.

#### Can institutional improvements and democratization cancel out the oil curse on FDI?

Yes, *ceteris paribus*, even if oil production overall hampers FDI, these barriers could be overcome through institutional reforms. As in [20], we calculate the average level of institutional quality and democratization necessary to cancel out the oil curse on FDI. This threshold is computed as follows:

$$\begin{array}{c} \frac{\partial ln(FDI)}{\partial oil}={\hat{\beta }}_{10}+{\hat{\beta }}_{12}{\bar{Inst}}_{threshold}=0;{\bar{Inst}}_{threshold}=-\frac{{\hat{\beta }}_{10}}{{\hat{\beta }}_{12}} \end{array}$$ (3)

The results are reported in Table 6, where examples are also provided. The institutional improvements required to cancel out the negative impact oil dependence has on FDI would imply attaining levels of other developing countries such as the Republic of Macedonia, Georgia or China. The progress required is attainable; for countries like Algeria, Nigeria and Russia an increase inferior to one point in the indices would be sufficient to ensure that oil dependence does not hamper FDI. In addition, based on the estimates from Table 4, these institutional reforms could boost new greenfield projects in Algeria, Nigeria and Russia by 14.32%, 23.88% and 15.56%, respectively.

**Table 6:** Institutional quality and democracy levels required to counterbalance the oil curse on FDI.

	Level required to cancel out the oil curse	As in	Country with lower institutional quality	Improvement in institution required to cancel out the oil curse
**Rule of law**	3.29	Republic of Macedonia	Algeria	0.32
**Lack of corruption**	2.63	Georgia	Nigeria	0.80
**Political stability**	3.80	China	Russia	0.49

Note: Authors’ own calculations, based on estimates from Table 4 and the average level of institutions of countries during the period 2003-2012. As in the econometric analysis, institutional variables are converted in a way that they equal or are larger than 1.

## Robustness and sensitivity analysis

In the following lines, we perform a battery of robustness checks to support our analysis. Table 3 may suffer from an omitted variable bias since we do not control for the pure effect that oil abundance may have on FDI. Then, the robustness and sensitivity analysis focus on the specification from Table 4. To conserve space, we only comment on the key estimates, namely the coefficients associated to oil production and institutional quality.

### Alternative measures of oil abundance

We test the sensitivity of our results to different measures of oil production. Following [11, 20] and [9], we consider the share of fuel and minerals in merchandise exports (*FuelExports*_*jt*_) as an alternative measure of oil abundance. In addition, we also test whether the previous findings hold when considering countries’ share of World oil production (*OilShare*_*jt*_) or the absolute number of oil-barrels production (*OilBarrels*_*jt*_). The share of fuels and minerals in merchandise exports are retrieved from World Bank’s Development Indicators, and the number of oil barrels production from Thomson Reuters Eikon. Estimates are respectively available in Tables 7, 8 and 9. Our conclusions remain basically unchanged. They validate the oil curse on greenfield investment projects, and show that the positive effect of institutional quality is enhanced by the level of oil abundance. Furthermore, the interaction of the natural resource measure and democracy becomes positive and significant.

**Table 7:** Institutions and share of fuel in merchandise exports, extensive margin.

FDI	Rule of law	Lack of corruption	Political stability	Democracy
Extensive margin	(1)	(2)	(3)	(4)
*FuelExports*_*jt*_	-0.041***	-0.015**	0.017*	-0.001
	(0.01)	(0.01)	(0.01)	(0.00)
*Inst*_*jt*_	-0.079	-0.120	0.248***	0.005
	(0.14)	(0.08)	(0.06)	(0.01)
*Inst*_*jt*_*xFuelExports*_*jt*_	0.015***	0.010***	-0.001	0.001***
	(0.00)	(0.00)	(0.00)	(0.00)
Fixed effects	Home, host and year fixed effects			
Control variables	YES	YES	YES	YES
Observations	35851	35851	35851	34223
*R*^2^	0.852	0.852	0.851	0.851

Robust standard errors in parentheses

* *p* < 0.10,

** *p* < 0.05,

*** *p* < 0.01

Coefficients of control variables are available on request.

**Table 8:** Institutions and oil barrels production share of world’s output.

FDI	Rule of law	Lack of corruption	Political stability	Democracy
Extensive margin	(1)	(2)	(3)	(4)
*OilShare*_*jt*_	-0.722***	-0.143**	-0.218**	-0.274***
	(0.13)	(0.07)	(0.09)	(0.06)
*Inst*_*jt*_	-0.01	-0.094	0.180***	-0.002
	(0.13)	(0.09)	(0.05)	(0.01)
*OilShare*_*jt*_*xInst*_*jt*_	0.147***	0.037***	0.037**	0.018***
	(0.03)	(0.01)	(0.02)	(0.00)
Fixed effects	Home, host and year fixed effects			
Control variables	YES	YES	YES	YES
Observations	35632	35878	35877	34017
*R*^2^	0.846	0.847	0.850	0.850

Robust standard errors in parentheses

* *p* < 0.10,

** *p* < 0.05,

*** *p* < 0.01

Coefficients of control variables are available on request.

**Table 9:** Institutions and oil barrels, extensive margin.

FDI	Rule of law	Lack of corruption	Political stability	Democracy
Extensive margin	(1)	(2)	(3)	(4)
*OilBarrels*_*jt*_	-0.916***	-0.234***	-0.395***	-0.404***
	(0.17)	(0.08)	(0.13)	(0.09)
*Inst*_*jt*_	-0.019	-0.116	0.163***	-0.003
	(0.14)	(0.10)	(0.05)	(0.01)
*Inst*_*jt*_*xOilBarrels*_*jt*_	0.190***	0.062***	0.065***	0.027***
	(0.04)	(0.02)	(0.03)	(0.01)
Fixed effects	Home, host and year fixed effects			
Control variables	YES	YES	YES	YES
Observations	35877	35878	35878	34017
*R*^2^	0.848	0.847	0.850	0.851

Robust standard errors in parentheses

* *p* < 0.10,

** *p* < 0.05,

*** *p* < 0.01

Coefficients of control variables are available on request.

### Greenfield investment volume

The first robustness check tests whether the findings reached for the extensive margin are also extendable to the intensive margin. Thus, the dependent variable (number of greenfield investment projects) in Eq 1 is substituted by the total investment volume. Table 10 illustrates that only democracy appears to have a positive significant impact, while the remaining indicators and all the interactions are not significant. Furthermore, we do not find evidence supporting the hypothesis that oil production affects the amount of greenfield investments.

**Table 10:** Institutions and oil rents over GDP, intensive margin.

FDI	Rule of law	Lack of corruption	Political stability	Democracy
Intensive margin	(1)	(2)	(3)	(4)
*ln*(*GDP*_*it*_*xGDP*_*jt*_)	-0.023	0.015	-0.047	-0.048
	(0.23)	(0.23)	(0.23)	(0.23)
*ln*(*Distance*_*ij*_)	-0.390***	-0.389***	-0.388***	-0.387***
	(0.06)	(0.06)	(0.06)	(0.07)
*BORDER*_*ij*_	-0.009	-0.010	-0.010	-0.010
	(0.14)	(0.14)	(0.14)	(0.14)
*LANGUAGE*_*ij*_	0.481***	0.481***	0.481***	0.479***
	(0.11)	(0.11)	(0.11)	(0.12)
*COLONY*_*ij*_	0.461***	0.462***	0.462***	0.459***
	(0.11)	(0.11)	(0.11)	(0.11)
*SMCNTRY*_*ij*_	0.398	0.399	0.399	0.384
	(0.26)	(0.26)	(0.26)	(0.26)
*Religion*_*ij*_	0.836***	0.837***	0.837***	0.834***
	(0.23)	(0.23)	(0.23)	(0.24)
*FTA*_*ijt*_	0.160	0.164	0.165	0.168
	(0.11)	(0.11)	(0.11)	(0.11)
*BIT*_*ijt*_	-0.102	-0.103	-0.101	-0.095
	(0.07)	(0.07)	(0.07)	(0.08)
*OilRents*_*jt*_	-0.012	-0.036	0.017	0.029
	(0.04)	(0.04)	(0.03)	(0.02)
*Inst*_*jt*_	-0.259	-0.214	0.095	0.050**
	(0.26)	(0.18)	(0.11)	(0.02)
*Inst*_*jt*_*xOilRents*_*jt*_	0.010	0.024	-0.000	-0.000
	(0.01)	(0.01)	(0.01)	(0.00)
Fixed effects	Home, host and year fixed effects			
Control variables	YES	YES	YES	YES
Observations	36037	36037	36004	34920
*R*^2^	0.434	0.434	0.433	0.435

Robust standard errors in parentheses

* *p* < 0.10,

** *p* < 0.05,

*** *p* < 0.01

### Unobserved and omitted variables

#### Country pair fixed effects

The second robustness check consists of testing whether the estimated models suffer from omitted variables in terms of the time-invariant transaction costs between pair of countries. To this end, in Eq 1, we substitute the bilateral time invariant variables and the source and host countries fixed effects by country pair fixed effects (λ_*ij*_). Estimates for the extensive and intensive margin are available in Table 11. As can be seen, in both margins, the significance, sign and size of the coefficients remain unchanged.

**Table 11:** Institutions and oil rents over GDP with country pair fixed effects.

	Rule of law		Lack of corruption		Political stability		Democracy	
	FDIp	FDIv	FDIp	FDIv	FDIp	FDIv	FDIp	FDIv
	(1)	(2)	(3)	(4)	(5)	(6)	(7)	(8)
*OilRents*_*jt*_	-0.056***	-0.014	-0.042***	-0.038	-0.019*	0.019	-0.000	0.032
	(0.01)	(0.03)	(0.01)	(0.04)	(0.01)	(0.03)	(0.01)	(0.02)
*Inst*_*jt*_	0.109	-0.209	-0.038	-0.196	0.244***	0.091	0.024***	0.061**
	(0.13)	(0.26)	(0.08)	(0.18)	(0.06)	(0.11)	(0.01)	(0.02)
*Inst*_*jt*_*xOilRents*_*jt*_	0.017***	0.010	0.016***	0.025*	0.005**	-0.001	-0.000	-0.002
	(0.00)	(0.01)	(0.00)	(0.01)	(0.00)	(0.01)	(0.00)	(0.00)
Fixed effects	Country pair and year fixed effects							
Control variables	Yes	Yes	Yes	Yes	Yes	Yes	Yes	Yes
Observations	36016	36016	36016	36016	36010	36010	34819	34819
*R*^2^	0.901	0.719	0.901	0.720	0.901	0.719	0.905	0.726

Robust standard errors in parentheses

* *p* < 0.10,

** *p* < 0.05,

*** *p* < 0.01

FDIp is the extensive margin and FDIv is the intensive margin. Coefficients of control variables are available on request.

#### Migration stock

Recent literature has stressed the influence of migration on FDI and particularly on greenfield FDI [76, 77]. The diaspora of MENA country migrants in particular, and oil-producing countries in general, is non-negligible, and therefore might be a source of omitted variable bias. To test for this, we have introduced the bilateral stock of migrants (taken from the OECD migration database) in our regressions. The data of migrants restricts the source countries to OECD countries. However, the results reported in Table 12 confirm the robustness of our results considering both out of sample validity and omitted variable bias in relation to migrant stocks.

**Table 12:** Institutions and oil rents over GDP with bilateral migration.

	Rule of law		Lack of corruption		Political stability		Democracy	
	FDIp	FDIv	FDIp	FDIv	FDIp	FDIv	FDIp	FDIv
	(1)	(2)	(3)	(4)	(5)	(6)	(7)	(8)
*Migration*_*ji*_	-0.020	0.033	-0.019	0.038	-0.020	0.035	-0.028	0.010
	(0.03)	(0.09)	(0.03)	(0.09)	(0.03)	(0.09)	(0.03)	(0.10)
*OilRents*_*jt*_	-0.041**	-0.048	-0.041***	-0.069	-0.056***	-0.038	-0.020**	0.041
	(0.02)	(0.05)	(0.01)	(0.04)	(0.02)	(0.03)	(0.01)	(0.03)
*Inst*_*jt*_	0.173	0.134	0.035	-0.087	0.207***	0.194	0.016*	0.039
	(0.17)	(0.36)	(0.11)	(0.24)	(0.08)	(0.14)	(0.01)	(0.02)
*Inst*_*jt*_*xOilRents*_*jt*_	0.008	0.028	0.011*	0.046**	0.011**	0.023**	0.001	0.001
	(0.01)	(0.02)	(0.01)	(0.02)	(0.00)	(0.01)	(0.00)	(0.00)
Fixed effects	Country pair and year fixed effects							
Control variables	Yes	Yes	Yes	Yes	Yes	Yes	Yes	Yes
Observations	12146	12146	12146	12146	12146	12146	11717	11717
*R*^2^	0.937	0.781	0.937	0.781	0.937	0.781	0.939	0.785

Robust standard errors in parentheses

* *p* < 0.10,

** *p* < 0.05,

*** *p* < 0.01

FDIp is the extensive margin and FDIv is the intensive margin. Coefficients of control variables are available on request.

### Endogeneity issues

The fourth robustness check deals with the potential endogeneity between inward FDI and countries’ institutional quality and oil production. For instance, [90] suggest that total FDI can influence countries’ institutional quality or degree of democracy. Similarly, by providing the necessary capital and technology, MNEs can facilitate countries’ oil production (e.g. [1, 12]). In this regard, it is important to highlight three relevant aspects of the present analysis. First, the endogeneity issue between institutional quality and FDI is a lesser concern when explaining bilateral investment projects. Contrary to the case of total inward FDI, the investments from only one country are less likely to affect the host countries’ institutional framework (see [91]). Second, during our period of analysis, only 1.8% of the world’s greenfield investment projects reached the mining, quarrying and petroleum sector (Shares are calculated by the authors based on the annex Table 23 from [17]). Third, oil rents are the difference between the value of crude oil production and total costs of production. Consequently, oil rents do not account for rents obtained by extraction companies [12]. Nevertheless, countries’ quality of governance or level of oil production might not be fully exogenous to bilateral FDI.

To address whether this potential endogeneity bias affects the baseline results, we estimate two alternative specifications of Eq 1. First, following [42] and [12], we lag in one period the independent variables that refer to institutions and oil production. Results of the extensive and intensive margin are reported in Table 13. Estimates of the extensive margin corroborate that the benefits from improving institutional quality and the level of democracy is enhanced by the level of oil production. Similarly, the results of the intensive margin are identical to those reported in Table 10.

**Table 13:** Institutions and oil rents over GDP with lagged variables.

	Rule of law		Lack of corruption		Political stability		Democracy	
	FDIp	FDIv	FDIp	FDIv	FDIp	FDIv	FDIp	FDIv
	(1)	(2)	(3)	(4)	(5)	(6)	(7)	(8)
*OilRents*_*jt*−1_	-0.039***	-0.000	-0.047***	-0.018	-0.028***	-0.015	0.010*	0.014
	(0.01)	(0.03)	(0.01)	(0.03)	(0.01)	(0.03)	(0.01)	(0.01)
*Inst*_*jt*−1_	0.129	-0.039	-0.156*	-0.347	0.268***	0.207*	0.008	0.045*
	(0.13)	(0.25)	(0.09)	(0.24)	(0.06)	(0.11)	(0.01)	(0.02)
*Inst*_*jt*_*xOilRents*_*jt*−1_	0.015***	0.001	0.022***	0.008	0.009***	0.004	-0.000	-0.001
	(0.00)	(0.01)	(0.00)	(0.01)	(0.00)	(0.01)	(0.00)	(0.00)
Fixed effects	Home, host and year fixed effects							
Control variables	Yes	Yes	Yes	Yes	Yes	Yes	Yes	Yes
Observations	29912	29912	29912	29912	29843	29843	29046	29046
*R*^2^	0.853	0.425	0.854	0.424	0.860	0.429	0.854	0.427

Robust standard errors in parentheses

* *p* < 0.10,

** *p* < 0.05,

*** *p* < 0.01

FDIp is the extensive margin and FDIv is the intensive margin. Coefficients of control variables are available on request.

The second specification replicates that of [11] but applied to bilateral FDI as in [92]. We estimate a linear dynamic panel data model with the system Generalised Method of Moments (GMM) proposed by [93]. In this way, Eq 1 includes as independent variable the lagged FDI, and bilateral time-invariant variables are replaced with country pair fixed effects. We use the two-step system GMM estimator, and specify oil production (*OilRents*_*jt*_) and institutions (*Inst*_*jt*_) variables as endogenous, while the remaining independent variables are considered as strictly exogenous. In line with [11], our estimations use the lagged levels of all the independent variables as instruments. Therefore, the system GMM estimator also deals with the endogeneity of other potential problematic independent variables [94].

Estimates are presented in Table 14. In both margins, the lagged value of FDI is positive as expected but with low values since FDI exhibits a less persistent behaviour than trade. As it can be gathered, evidence of a oil resource curse on FDI is reached for both margins. Furthermore, on the extensive margin, the results ratify that the benefits of improving rule of law and reducing corruption are magnified by oil endowment. Similar conclusions are reached on the intensive margin for rule of law, lack of corruption and political stability.

**Table 14:** Institutions and oil rents over GDP: System GMM estimate.

	Rule of law		Lack of corruption		Political stability		Democracy	
	FDIp	FDIv	FDIp	FDIv	FDIp	FDIv	FDIp	FDIv
	(1)	(2)	(3)	(4)	(5)	(6)	(7)	(8)
*FDI*_*ijt*−1_	0.093***	0.059***	0.084***	0.061***	0.091***	0.060***	0.090***	0.062***
	(0.01)	(0.01)	(0.01)	(0.01)	(0.01)	(0.01)	(0.01)	(0.01)
*OilRents*_*jt*_	-0.001	-0.011**	-0.005***	-0.034***	0.000	-0.010**	-0.002	0.004
	(0.00)	(0.01)	(0.00)	(0.01)	(0.00)	(0.00)	(0.00)	(0.00)
*Inst*_*jt*_	0.055***	0.157***	0.006	-0.010	0.077***	0.192***	-0.001	0.025**
	(0.02)	(0.06)	(0.02)	(0.05)	(0.02)	(0.06)	(0.00)	(0.01)
*Ins*_*jt*_*txOilRents*_*jt*_	0.000	0.003*	0.003***	0.015***	0.000	0.002**	0.000**	-0.000
	(0.00)	(0.00)	(0.00)	(0.00)	(0.00)	(0.00)	(0.00)	(0.00)
Fixed effects	Country pair and year fixed effects							
Control variables	Yes	Yes	Yes	Yes	Yes	Yes	Yes	Yes
Observations	32580	32580	32580	32580	32558	32558	31523	31523

Robust standard errors in parentheses

* *p* < 0.10,

** *p* < 0.05,

*** *p* < 0.01

FDIp is the extensive margin and FDIv is the intensive margin. Coefficients of control variables are available on request.

## Conclusions

The present article, by estimating a gravity equation, addresses how oil abundance, institutions and the interaction between both affects countries’ capacity to foster greenfield investment. To this end, we exploit a greenfield investment bilateral database which covers 182 countries during the period 2003-2012. We use alternative measures of oil production to take into account the dependence of the host on oil production and the dependence of the world on the host’s production. Moreover, we tackle institutions in a broad manner by considering rule of law, corruption, political stability and democracy.

According to our results, institutional quality and democracy appear to be a crucial dimension in defining significant oil-producing countries’ capacity for attracting new greenfield projects. Regarding a possible “oil curse” on FDI, our results confirm that overall, oil-abundant countries attract fewer greenfield projects than others. In addition, the evidence obtained suggests that countries with better governance and more democracy would attract more greenfield investments, with this effect being larger for countries highly dependent on oil and for main players in the world oil market. Thus, for oil producers, institutional reforms can significantly improve their capacity for attracting new investment partners. These reforms may raise the opportunity to diversify their economy and reduce the likelihood of suffering from the oil curse on FDI.

Our conjecture for this apparently puzzling result is that, when national production is heavily dependent on oil, the government might well be heavily dependent on these resources but may lack the capital to exploit these resources, which makes governments more willing to attract foreign projects. For these countries with high economic dependence on oil but with the lack of capital to exploit it, institutional reforms are likely to increase their capacity to attract foreign capital. When the host-country production represents a significant share of the world’s output, the host government is empowered, allowing it to sustain closed-economy policies combined with rent-seeking behaviour by the domestic oligarchy and does not need foreign investors. [30] argue that countries with large reserves of oil and gas have enough financial resources and foreign currency available to finance their own economic development. They may prefer to contract expertise services rather than incentivise FDI. Oil-rich countries have typically not actively encouraged FDI and have stipulated local ownership requirements in many, if not all, industry sectors [95]. In this way, similar to the conclusions reached by [23] for the MENA region or [51] in the analysis of democracy, the overall improvement of institutional quality and democracy favours countries’ integration into the world economy. For those countries that enjoy an oligopolistic position in oil production, significant institutional reforms would imply withdrawing these barriers to FDI.

Our study offers also an interesting evidence-based policy implication for countries approaching FDI promotion. The specialized literature interprets a growth in the extensive margin as the effect of FDI creation through new investment partners. We find a robust negative effect of the oil variables on the extensive margin. This effect, however, is not so robust on the intensive margin (value of flows). These findings suggest that, while oil-abundant countries attract fewer investment projects than similar countries without oil, the total value of investment remains relatively unchanged. This result is compatible with a scenario where investment is highly concentrated on a group of countries in a particular sector, with a high dependency on foreign technology to exploit oil rents. This limits the policy-maker’s options to confront an economic downturn or technological disruption in their niche countries and sector. However, new research in this area is needed to further study this issue—for example, the effect of the progressive decarbonisation of advanced economies on potential investors.

## Supporting information

S1 Appendix Country classification. Raw Data File.
(PDF)
