# Supplementary material for: Foreign Direct Investment in oil-abundant countries: The role of institutions
Source: PLoS One. 2019 Apr 23;14(4):e0215650. doi: 10.1371/journal.pone.0215650 (PMC6478325; doi:10.1371/journal.pone.0215650)
Supplement: S1 Appendix — Raw Data File. (PDF) [file pone.0215650.s001.pdf]

Table A: **Country classification**

**Oil countries**	**Non-oil countries**	**Non-oil countries**
Algeria	Cape Verde	Maldives
Angola	Cayman Islands	Mali
Azerbaijan	Central African Republic	Malta
Brunei	Chile	Martinique
Cameroon	China	Mauritius
Chad	Colombia	Mexico
East Timor	Comoros	Moldova
Ecuador	Costa Rica	Monaco
Egypt	Côte d'Ivoire	Mongolia
Equatorial Guinea	Croatia	Montenegro
Gabon	Cuba	Morocco
Iran	Cyprus	Mozambique
Iraq	Czech Republic	Myanmar (Burma)
Kazakhstan	Democratic Republic of Congo	Namibia
Kuwait	Denmark	Nepal
Libya	Djibouti	Netherlands
Mauritania	Dominica	New Zealand
Nigeria	Dominican Republic	Nicaragua
Norway	El Salvador	Niger
Oman	Eritrea	Pakistan
Papua New Guinea	Estonia	Panama
Qatar	Ethiopia	Paraguay
Republic of the Congo	Fiji	Peru
Russia	Finland	Philippines
Saudi Arabia	France	Poland
Sudan	Gambia	Portugal
Suriname	Georgia	Romania
Syria	Germany	Rwanda
Trinidad and Tobago	Ghana	Saint Vincent and Grenadines
Turkmenistan	Greece	São Tomé and Príncipe
UAE	Greenland	Senegal
Venezuela	Grenada	Serbia
Vietnam	Guatemala	Seychelles
Yemen	Guinea	Sierra Leone
**Non-oil countries**	Guinea Bissau	Singapore
Afghanistan	Guyana	Slovakia
Albania	Haiti	Slovenia
Antigua and Barbuda	Honduras	Somalia
Argentina	Hong Kong	South Africa
Armenia	Hungary	South Korea
Australia	Iceland	Spain
Austria	India	Sri Lanka
Bahamas	Indonesia	St Lucia
Bahrain	Ireland	Swaziland
Bahrain	Israel	Sweden
Bangladesh	Italy	Switzerland
Barbados	Jamaica	Taiwan
Belarus	Japan	Tajikistan
Belgium	Kenya	Tanzania
Belize	Kyrgyzstan	Thailand
Benin	Laos	Togo
Bermuda	Latvia	Tunisia
Bhutan	Lebanon	Turkey
Bolivia	Lesotho	Uganda
Bosnia-Herzegovina	Liberia	Ukraine
Botswana	Lithuania	United Kingdom
Brazil	Luxembourg	United States
Bulgaria	Macau	Uruguay
Burkina Faso	Macedonia FYR	Uzbekistan
Burundi	Madagascar	Zambia
Cambodia	Malawi	Zimbabwe
Canada	Malaysia	

Oil countries are those in which in at least one year oil rents represented 7.65% of GDP.
